# The Attitude of South Korean People Regarding Usage of The Internet Perinatal Consultation

**Published:** 2014-11-01

**Authors:** Tae-Hee Kim Kim, Hae-Hyeog Lee Lee, Soo-Ho Chung

**Affiliations:** Department of Obstetrics and Gynecology, Soonchunhyang University College of Medicine, Bucheon, Republic of Korea

**Keywords:** Delivery, Internet, Pregnancy, Perinatal care

## Abstract

**Background:**

To the general public, the Internet is an acceptable method of obtaining information. It also plays an important role in guiding patients and solving their problems. We investigated the clinical characteristics of an Internet website to provide guidelines and tips for consultation.

**Materials and Methods:**

In this retrospective observational study, we analyzed the use of a free public Internet perinatal consultation website provided by the Ministry of Health and Welfare of Korea. We evaluated 2,254 Internet perinatal consultations and assessments of prenatal and obstetrics from August 2006 to December 2009. We evaluated the patients’ questions based on Williams’ textbook categories and their clinical characteristics.

**Results:**

The mean age of patients seeking consultation was 33.9 ± 13.2 years, and parity was 1.2 ± 0.5. The most commonly asked questions were about drug safety during pregnancy (20.4%), and questions regarding prenatal care (18.7%) and labor pain (15.4%) were the second and third most commonly asked questions, respectively.

**Conclusion:**

The Internet can provide good information to patients. Thus, guidelines regarding pregnancy-related questions and answers should be created. Obstetricians could use our data to identify question tendencies.

## Introduction

Women who wish to become pregnant usually seek educational methods such as books and magazines for safe fertility and delivery. The Internet is currently a common and acceptable method by which patients seek answers to their questions. Based on 33 websites about Internet usage by pregnant women, 96.6% of women have Internet access at home (1). Pregnant women are often very nervous and curious about changes that occur to their body. However, some cannot easily get in touch with a doctor for counseling. Thus, women desire an access method not only for education, but also to easily and quickly obtain answers.

In the present study, we investigated the clinical characteristics of questions on an Internet website to provide guidelines and tips for consultation.

## Materials and Methods

In this retrospective observational study, we analyzed the use of a free public Internet perinatal consultation website provided by the Ministry of Health and Welfare of Korea. This website provides information on pregnancy, delivery, child care, and infant health programs and provides a multilingual system for immigrants as well.

The website also offers perinatal consultations and assessments of gynecologic problems free of charge. On average, there were 25,192 logins per day. The service was accessible via a Korean web domain. Fourteen obstetricians conducted the consultations using a private personal computer. Pediatric doctors also consulted with patients about pediatric problems. The consultant had a duty to reply to each patient’s questions. Women asked questions on the site, and an automatic, immediate, repetitive alarm sounded on the consultant’s telephone until a reply was updated on the website. The consultant was required to complete the reply while on duty. Duty days covered all 365 days of the year. All replies were completed within 24 hours.

We evaluated 2,254 consultations from August 2006 to December 2009. A total of 122 consultations occurred during the initiation year, whereas 802 consultations were performed in 2009. Two research nurses and one obstetrician evaluated each consultation. We evaluated the questions based on Williams’ textbook categories and patients’ clinical characteristics.

### Ethical considerations

Approval for this study was given by the Human Ethics Committee at SCH (Soonchunhyang University) Medical Center (Bucheon, Korea). We mention that no personal data is published and the privacy of the users was respected.

### Statistical analysis

Results are presented as number and percentage values. Variables were expressed using the number and the percentage. Statistical data were generated with the SPSS version 12 for Windows (SPSS Inc., Chicago IL, USA).

## Results

**Fig 1:**
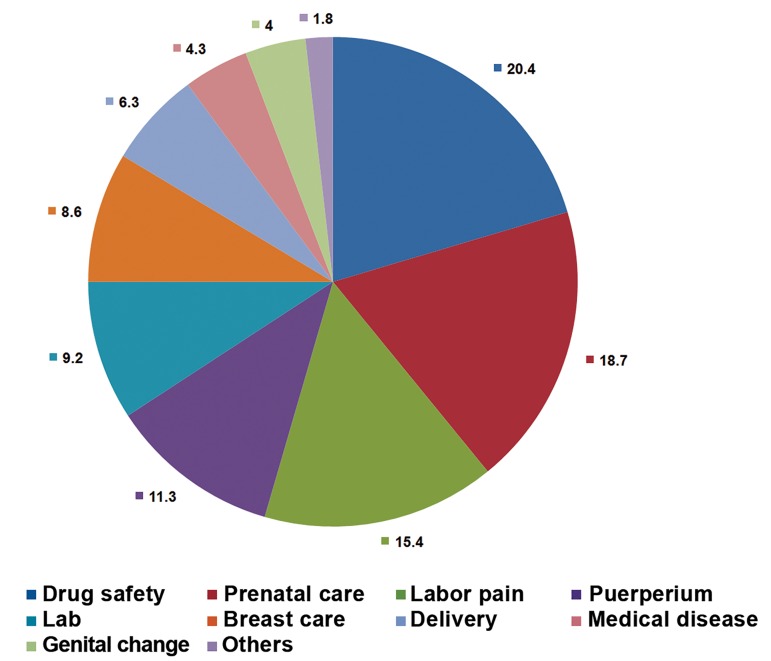
Percentage of asked questions by subject.

The mean age of patients seeking consultation was 33.9 ± 13.2 years, and parity was 1.2 ± 0.5. Most of the consultations were with women (2,125, 94.3%) (Table 1), and the greatest proportion of consultations (73.6%) were with women aged 25 to 35 years (Table 2). A total of 1,997 (88.6%) married women received consultations (Table 3). The residential distribution was as follows: Seoul and the central area, 55.1%; other provinces, 39.4%; and foreign countries, 2.2% (Table 4). Approximately 60.7% of the patients who received a consultation did not want their consultations to be publicly shared (Table 5). The consultations according to trimester were first trimester, 24.6%; second, 19.2%; third, 12.8%; and postpartum, 17.7%. A total of 25.2% of the consultations were performed during the preconception period (Table 6). All of the Internet consultations were performed in the Korean language. We divided the questions based on Williams’ textbook categories (2). The most commonly asked question was about drug safety during pregnancy (20.4%), and questions regarding prenatal care (18.7%) and labor pain (15.4%) were the second and third most commonly asked questions, respectively. Approximately 11.3% of the questions concerned postpartum care. Questions about laboratory interpretations during pregnancy, such as the quad test and breastfeeding methods or complications constituted 9.2% and 8.6% of questions, respectively. Labor and delivery questions, such as questions about induction or cesarean section, comprised 6.3% of the questions. Questions regarding hypertension and diabetes were raised at prevalence rates of 4.3% and 6.3%, respectively (Fig 1).

**Table 1:** Sex distribution of clients


Sex	Number	Percentage

**Male**	104	4.6
**Female**	2125	94.3
**Missed data**	25	1.1
**Total**	2254	100


**Table 2:** Age distribution of clients


Age	Number	Percentage

**< 25**	113	5.0
**25-35**	1660	73.6
**36-45**	396	17.6
**>45**	68	3.0
**Missed data**	17	0.8
**Total**	2254	100


**Table 3:** Marital status of clients


Marital status	Number	Percentage

**Married**	1997	88.6
**Unmarried**	186	8.3
**Missed data**	71	3.1
**Total**	2254	100


**Table 4:** Residential distribution of clients


Area	Number	Percentage

**Seoul and Gyeonggi**	1241	55.1
**Other provinces**	889	39.4
**Foreign country**	50	2.2
**Missed data**	74	3.3
**Total**	2254	100


**Table 5:** Agreement to publicly share information


Agreement	Number	Percentage

**Yes**	870	38.6
**No**	1368	60.7
**Missed data**	16	0.7
**Total**	2254	100


**Table 6:** Gestational period of clients by trimester


G.A.^*^ by trimester	Number	Percentage

**Pregestation**	567	25.2
**First**	554	24.6
**Second**	432	19.2
**Third**	288	12.8
**Postpartum**	398	17.7
**Missed data**	17	0.8
**Total**	2254	100


*; Gestational age.

## Discussion

In Korea, there are almost 80 known websites about pregnancy, delivery, and child care (3). Our dataset is the largest in Korea and is representative of Korea, as the evaluated website is supported by the Ministry of Health and Welfare of Korea. Therefore, our data are representative of the characteristics of pregnancy-related questions.

The Internet is useful for pregnant women and plays a role in decision-making. In a Web survey of Internet use in 24 countries, most women (97%) used the Internet for pregnancy-related questions (1). Mankata described hospital and physician referral as the most prevalent topic (15%) for Internet consultation (4). However, our data revealed that Korean pregnant women were most curious about drug safety during pregnancy (20.4%).

The preconception period was also a common topic of consultation based on our data. Mankata reported that 5% of those asking questions were husbands, similar to our study (4.7%). Women in other countries were interested in and had many questions regarding pregnancy, but the topics in which they were interested were not the same. Pregnancy-associated questions are common on the Internet, which is a very cost-effective way to obtain information (4). However, a review article about Internet consultations revealed that most studies on this topic have provided little evidence and have been poorly designed (5).

## Conclusion

The Internet can provide good information, but that information is sometimes confusing and may be inaccurate. If the website evaluated in this study had an automatic system for differentiating questions based on keywords, trimester, previous related questions, and so forth, the questions could easily be categorized and the evaluation time could be reduced. Thus, such a system should be created for pregnancy-related questions and answers. Culture and health care systems might influence the nature of the most frequent questions. We inferred that Korean women are very anxious about the health of the baby, so they were very eager to obtain information about drug safety and preconception care.
